# Deep learning for real-time detection of breast cancer presenting pathological nipple discharge by ductoscopy

**DOI:** 10.3389/fonc.2023.1103145

**Published:** 2023-03-22

**Authors:** Feng Xu, Chuang Zhu, Zhihao Wang, Lei Zhang, Haifeng Gao, Zhenhai Ma, Yue Gao, Yang Guo, Xuewen Li, Yunzhao Luo, Mengxin Li, Guangqian Shen, He Liu, Yanshuang Li, Chao Zhang, Jianxiu Cui, Jie Li, Hongchuan Jiang, Jun Liu

**Affiliations:** ^1^ Department of Breast Surgery, Beijing Chao-Yang Hospital, Capital Medical University, Beijing, China; ^2^ School of Artificial Intelligence, Beijing University of Posts and Telecommunications, Beijing, China; ^3^ Breast Disease Prevention and Treatment Center, Haidian Maternal and Child Health Hospital, Beijing, China; ^4^ Department of Breast Surgery, The Second Hospital of Dalian Medical University, Dalian, China; ^5^ Department of General Surgery , Beijing Huairou Hospital, Beijing, China; ^6^ Department of Breast Surgery, Beijing Yanqing District Maternal and Child Health Care Hospital, Beijing, China; ^7^ Department of General Surgery, Beijing Pinggu Hospital, Beijing, China

**Keywords:** breast cancer, deep learning, pathological nipple discharge, ductoscopy, diagnosis

## Abstract

**Objective:**

As a common breast cancer-related complaint, pathological nipple discharge (PND) detected by ductoscopy is often missed diagnosed. Deep learning techniques have enabled great advances in clinical imaging but are rarely applied in breast cancer with PND. This study aimed to design and validate an Intelligent Ductoscopy for Breast Cancer Diagnostic System (IDBCS) for breast cancer diagnosis by analyzing real-time imaging data acquired by ductoscopy.

**Materials and methods:**

The present multicenter, case-control trial was carried out in 6 hospitals in China. Images for consecutive patients, aged ≥18 years, with no previous ductoscopy, were obtained from the involved hospitals. All individuals with PND confirmed from breast lesions by ductoscopy were eligible. Images from Beijing Chao-Yang Hospital were randomly assigned (8:2) to the training (IDBCS development) and internal validation (performance evaluation of the IDBCS) datasets. Diagnostic performance was further assessed with internal and prospective validation datasets from Beijing Chao-Yang Hospital; further external validation was carried out with datasets from 5 primary care hospitals. Diagnostic accuracies, sensitivities, specificities, and positive and negative predictive values for IDBCS and endoscopists (expert, competent, or trainee) in the detection of malignant lesions were obtained by the Clopper-Pearson method.

**Results:**

Totally 11305 ductoscopy images in 1072 patients were utilized for developing and testing the IDBCS. Area under the curves (AUCs) in breast cancer detection were 0·975 (95%CI 0·899-0·998) and 0·954 (95%CI 0·925-0·975) in the internal validation and prospective datasets, respectively, and ranged between 0·922 (95%CI 0·866-0·960) and 0·965 (95%CI 0·892-0·994) in the 5 external validation datasets. The IDBCS had superior diagnostic accuracy compared with expert (0.912 [95%CI 0.839-0.959] vs 0.726 [0.672-0.775]; p<0.001), competent (0.699 [95%CI 0.645-0.750], p<0.001), and trainee (0.703 [95%CI 0.648-0.753], p<0.001) endoscopists.

**Conclusions:**

IDBCS outperforms clinical oncologists, achieving high accuracy in diagnosing breast cancer with PND. The novel system could help endoscopists improve their diagnostic efficacy in breast cancer diagnosis.

## Introduction

Breast cancer (BC) accounts for 24.2% of all cancers diagnosed in women worldwide, constituting the first female cancer (1). Pathological nipple discharge (PND) represents a common BC-related complaint (2). Compared to other imaging methods such as sonography, mammography and MRI, ductoscopy is currently the only intuitive and effective technique for clinical screening and diagnosis of BC because it allows for direct visualization of intraductal lesions that cause PND (3). Meanwhile, ductoscopy solves the problem of intraductal lesion localization and reduces the scope of surgery in most PND cases. Additionally, some surgical indications for PND have been revised, avoiding unnecessary surgery in some patients. However, early BC with PND often lacks typical endoscopic features leading to a missed diagnosis. Besides, intraductal biopsy under ductoscopy makes it difficult to diagnose the tumor histologically without surgery (4). On the other hand, there is a huge deficit of endoscopists in China, whose number is far from meeting the actual clinical needs. In addition, endoscopists in different levels of hospitals have distinct levels of expertise. As a result, there is a low detection rate for breast cancer with PND, which seriously affects the prognosis and aggravates the economic pressure on patients.

Deep-leaning (DL) methods have been utilized more commonly compared with other traditional machine-learning techniques (5). DL approaches have an outstanding capability of retracting visual properties of objects, even those not detectable by humans, and quickly analyzing large datasets (6, 7). DL-based approaches are increasingly applied to real-time computer-aided diagnosis (CAD) systems in gastrointestinal endoscopy (8–10). Mounting evidence reveals advantages for DL CAD models in detecting and characterizing diverse cancerous tumors (11, 12), at all levels of the gastrointestinal tract (13). To improve the diagnosis of intraductal lesions, especially BC, by ductoscopy, we aim to design tools that enhance real-time detection of intraductal cancers, providing guidance utilizing a pre-trained deep learning algorithm.

In this work, a deep learning model was designed for BC detection based on a fully convolutional network, called the Intelligent Ductoscopy for Breast Cancer Diagnostic System (IDBCS). The IDBCS and oncologists were comparatively assessed for diagnostic performance in internal test and prospective sets based on endoscopic images in patients administered routine ductoscopy screening for pathological nipple discharge. The IDBCS was next validated in other external validation sets in five municipal hospitals. The current study demonstrated that IDBCS had encouraging performance in distinguishing cancerous lesions. The novel IDBCS-based artificial intelligence platform could yield higher malignancy detection rates, thus improving patient survival.

## Methods

### Study design and patients

The present multicenter, case-control, diagnostic trial was carried out in 6 hospitals in China. Endoscopic images were retrospectively retrieved for the design and validation of an Intelligent Ductoscopy for Breast Cancer Diagnostic System (IDBCS) from the imaging database of Beijing Chao-Yang hospital (BCYH) between January 2018 and December 2020.

To generalize IDBCS applicability in clinic, endoscopic images were also retrieved from 5 municipal/provincial hospitals in China, including Beijing Haidian District Maternal and Child Health Care Hospital (HDH), the Second Hospital of Dalian Medical University (DLH), Beijing Huairou Hospital (HRH), Beijing Pinggu Hospital (PGH), and Beijing Yanqing District Maternal and Child Health Care Hospital (YQH).

All images were acquired at high-resolution but utilizing multiple endoscopes (FVY-680, Blade, China; Schoelly, German) and saved as jpeg files. Five endoscopists at BCYH, with at least 5 years of experience and >500 examinations performed, evaluated the image quality.

Inclusion criteria were: 1) PND with unilateral single duct; 2) postoperative histopathology; 3) complete baseline data, including age, duration of PND, characteristics of PND, color of nipple fluid, lesion base, intraductal location and morphology of tumor, and palpable mass; 4) ductoscopy carried out for pretreatment examination; 5) ductoscopy images with standard white light.

Exclusion criteria were: 1) PND during pregnancy or lactation; 2) incomplete postsurgical pathological data; 3) poor quality ductoscopy images such as narrow-band imaging, motion-blurring, blank and out-of-focus; 4) PND manifested as multiple ducts.

The present trial had approval from the respective institutional review boards of various participating hospitals, and followed the Helsinki declaration. Each patient assessed in the prospective validation dataset (BCYH) provided signed informed consent prior to enrolment. In individuals with endoscopic images stored in retrospective databases at various participating hospitals, the requirement for informed consent was waived by the respective institutional review boards.

### Development of the IDBCS algorithm

#### Dataset

Images from Chaoyang Hospital were divided into training and internal validation datasets according to a ratio of 8:2. To better train the AI model, image augmentation methods (14), e.g., horizontal and vertical flipping, were adopted. The training dataset was employed for model training, and the internal validation dataset was utilized to evaluate the model’s performance.

#### Overview

The IDBCS algorithm was developed based on a deep Convolutional Neural Network (15) (CNN), and achieved patient-level diagnosis using a voting mechanism. Specifically, the IDBCS algorithm consists of two stages. In the first stage, multiple images from the same patient are fed into the backbone (DenseNet) to obtain a series of positive probability scores. In the second stage, the model uses a voting mechanism where the positive probability scores of all images are averaged to obtain an average score, and when the average score exceeds a threshold, the patient is classified as positive.

#### Backbone

**Figure 1:**
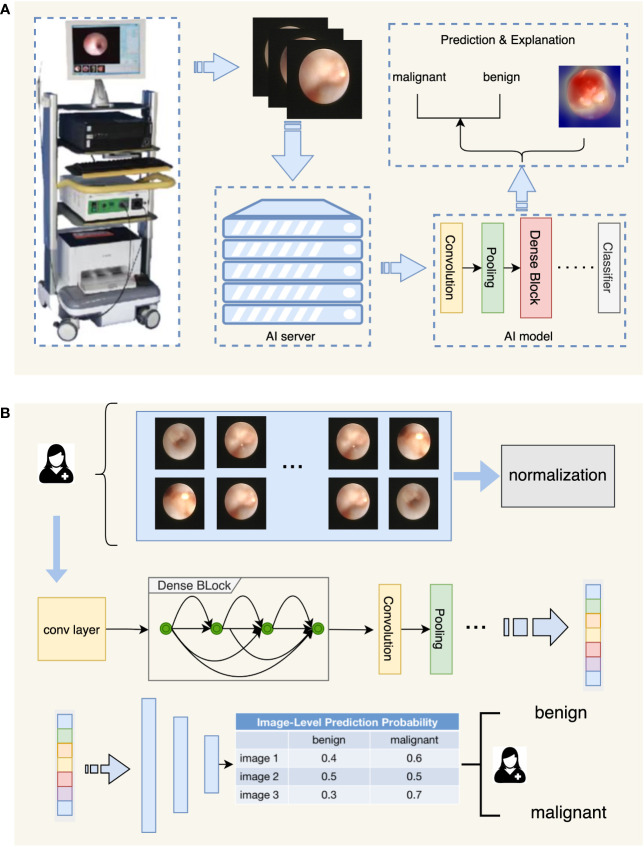
Flowchart depicting the diagnostic process of IDBCS. Part **(A)** in this figure shows the whole process of automatic diagnosis of ductoscopy images. The image obtained by the ductoscopy machine is transmitted to the server containing the AI algorithm in real-time, and the result is given after the automatic diagnosis of the AI algorithm, and the corresponding explanation is given for the diagnosis result. Part **(B)** in this figure shows the model structure of the diagnosing algorithm. Ductoscopy images were normalized first and then fed into the DenseNet model. DenseNet contains several Dense Blocks and calculates features of the input images. The output features were fed into the classifier with a voting mechanism and the diagnosing result was given.

During the development of the IDBCS algorithm, we applied four deep learning models, including VGG (16), Inception-v3 (17), ResNet (18), and DenseNet (19), which were all trained using the same dataset. DenseNet had the best performance in the validation dataset. Therefore, DenseNet was selected to develop IDBCS. DenseNet consists of multiple dense blocks and connects each layer to the others by feed-forward. In DenseNet, every layer takes supplemental input from the previous layers and transmits the extracted features to following layers. Thus, every layer can receive collective knowledge from the previous ones. The DenseNet structure solves the problem of vanishing gradient during the training process, reduces the number of parameters, and improves the inference speed while ensuring high performance, which matches the real-time diagnosis characteristics of IDBCS. (**Figure 1**).

#### Training

For each ductoscopy image, we scale the image to 224 pixels in length and width, and normalize the image with a mean set to [0.4914, 0.4822, 0.4465], variance set to [0.2023, 0.1994, 0.2010]. We train the model on Tesla T4 GPU with the learning rate set to 0.01 and the batch size set to 32.

### Validation of the IDBCS algorithm

We first used the internal validation and prospective datasets retrieved from Chaoyang Hospital to preliminarily assess the model’s performance. To further assess the model’s robustness and generalization, 5 external validation datasets from different hospitals were utilized for model testing.

For performance comparison between IDBCS and endoscopists, we invited 3 experts, 3 competent, and 3 trainees to diagnose 102 patients in the prospective Chaoyang dataset. Before the diagnosis, all 9 doctors had no information about the dataset to ensure the authenticity of the experiment.

### Statistical analysis

Diagnostic accuracy (ACC), sensitivity (SENS), specificity (SPEC), positive (PPV) and negative (NPV) predictive values, and the area under the receiver operating characteristic (20) (ROC) curve (AUC) were determined to evaluate the performance of the IDBCS in breast ductal tumor diagnosis.

All the metrics are calculated based on patient-level malignant probability according to the following equation.


(1) $$P_{patient}=average\left(p_{1},p_{2},p_{3}\ldots p_{n}\right)$$

where *p*_1_,*p*_2_,…,*p*_*n*_ are the malignant probability of all the images from the same patient. All the metrics are calculated according to the following equations.


(2) $$ACC=\frac{TP\;+\;TN}{TP\;+\;TN\;+\;FP\;+\;FN}\;$$

(3) $$SENS=\frac{TP}{TP+FN}$$

(4) $$SPEC=\frac{TN}{TN+FP}$$

(5) $$PPV=\frac{TP}{TP+FP}$$

(6) $$NPV=\frac{TN}{TN+FN}$$

where TP, TN, FP, and FN represent true positives, true negatives, false positives, and false negatives, respectively.

For the comparison of IDBCS and endoscopists for diagnostic performance, we collected the diagnostic results of expert, competent and trainee endoscopists. According to these diagnostic results, ACC, SENS, SPEC, PPV and NPV were calculated to assess the difference in diagnostic performance between IDBCS and endoscopists. Meanwhile, all statistics were two-sided, and 95% confidence intervals for various metrics were determined by the Bootstrap method (21). All continuous variables were compared by the t-test. All statistical analyses were performed with the MedCalc software (22) and python 3.7.

## Results

### Baseline features of the training and test datasets

**Figure 2:**
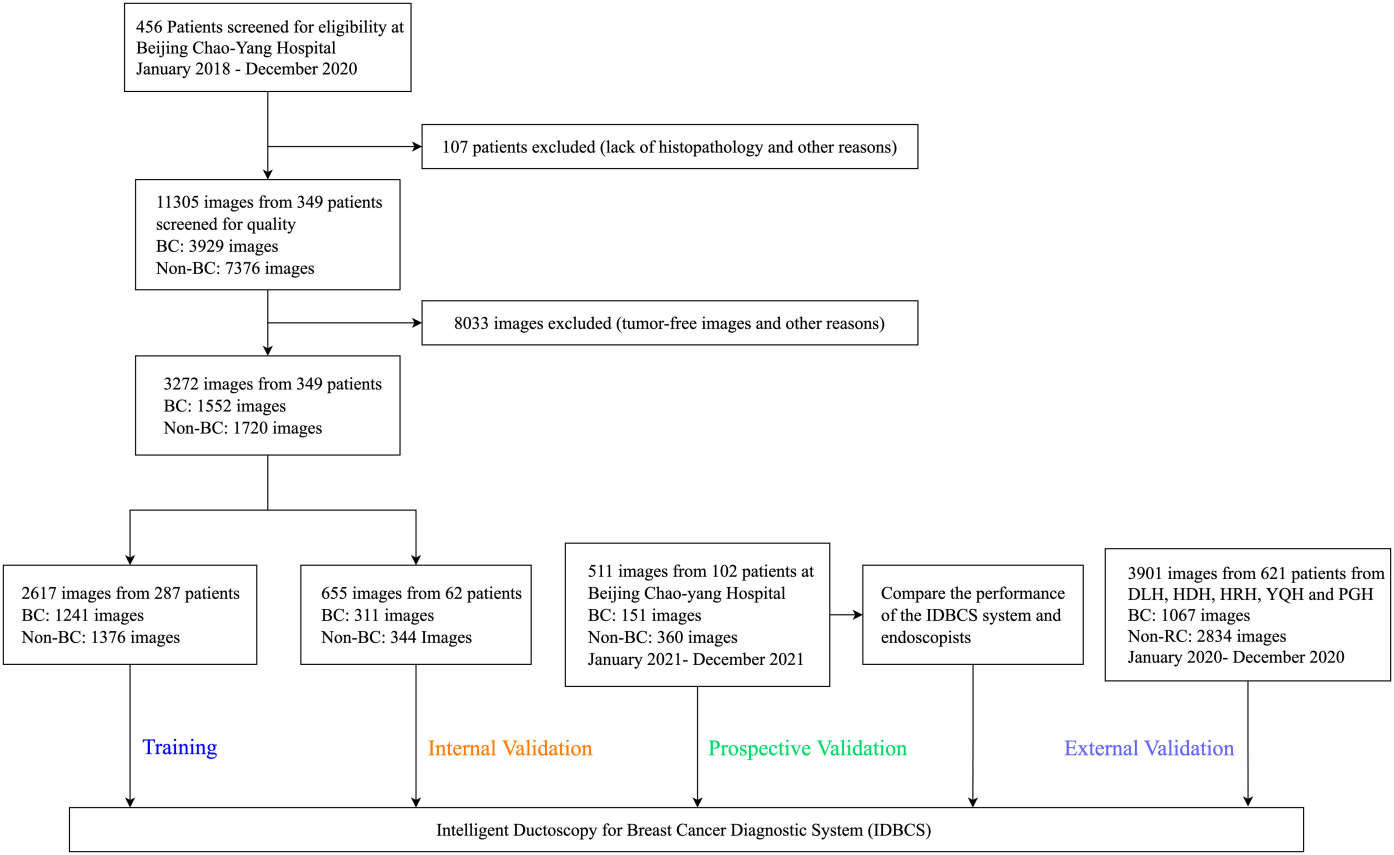
Flowchart for the development and validation of the IDBCS system for diagnosing breast cancer with pathological nipple discharge.

Between January 1, 2018, and December 31, 2020, 456 patients were treated at BCYH (**Figure 2**). Due to unknown pathological diagnosis and incomplete pathological data, 107 patients were excluded. Following quality control, 8033 images were excluded as tumor-free or poor-quality images. For cancer cases, only the images of cancerous tumors were examined (n=1552); for those with no malignancy, 1720 images were utilized as controls (**Figure 2**). In the prospective validation dataset, 151 cancerous tumors and 360 control images were prospectively obtained between January 1, 2021, and December 31, 2021.

At the other five participating hospitals, between January 1, 2020, and December 31, 2020, 793 cancer and 242 control images were obtained from DLH, 902 cancer and 400 control images from HDH, 583 cancer and 276 control images from HRH, 185 cancer and 50 control images from YQH, and 371 cancer and 99 control images from PGH. Overall, 7684 ductoscopy images in 1072 participants were utilized for IDBCS development and testing.

BC prevalence rates were 26.8% (77/287) in the training dataset, 29.0% (18/62) in the internal validation dataset, 31.4% (32/102) in the prospective validation dataset, 27.7% (41/148) in DLH, 34.1% (73/214) in HDH, 26.7% (32/120) in HRH, 22.4% (15/67) in YQH, and 23.6% (17/72) in PGH (**Table 1**).

**Table 1:** Baseline characteristics.

	BCYH training set		BCYH internal validation set		BCYH prospective test set		DLH test set		HDH test set		HRH test set		YQH test set		PGH test set	
	Non-BC	BC	Non-BC	BC	Non-BC	BC	Non-BC	BC	Non-BC	BC	Non-BC	BC	Non-BC	BC	Non-BC	BC
**Patients**	210	77	44	18	70	32	107	41	141	73	88	32	52	15	55	17
**Images**	1376	1241	344	311	360	151	793	242	902	400	583	276	185	50	371	99
Age (year)																
≤40	97	32	18	5	30	12	47	18	54	30	31	10	19	6	24	7
>40	113	45	26	13	40	20	60	23	87	43	57	22	33	9	31	10
Duration of PND (month)																
≤6	174	69	36	16	49	26	61	28	100	49	62	25	40	11	30	13
>6	36	8	8	2	21	6	46	13	41	24	26	7	12	4	25	4
PND characteristics																
Serous	182	50	37	10	60	15	88	20	108	40	69	20	44	10	45	13
Bloody	28	27	7	8	10	17	19	21	33	33	19	12	8	5	10	4
Spontaneous or not																
Spontaneous	120	46	34	15	43	23	84	32	111	58	68	17	28	8	36	9
Non-spontaneous	90	31	10	3	27	9	23	9	30	15	20	15	24	7	19	8
Color of PND																
Colorless	51	2	7	2	14	3	55	9	36	20	25	8	14	3	10	6
Yellow	131	48	30	8	46	12	33	11	72	20	44	12	30	7	35	7
Brown	18	17	5	7	7	13	9	11	16	18	13	8	6	3	4	1
Red	10	10	2	1	3	4	10	10	17	15	6	4	2	2	6	3
Ductal wall																
Smooth	124	37	32	7	42	13	80	18	60	33	51	16	38	7	40	10
Rough	86	40	12	11	28	19	27	23	81	40	37	16	14	8	15	7
Lesion base																
Broad	63	31	19	10	23	17	43	21	46	34	42	18	13	8	25	9
Narrow	147	46	25	8	47	15	64	20	95	39	46	14	39	7	30	8
Morphology																
Regular	142	33	27	6	51	14	81	16	90	32	53	15	30	6	34	8
Irregular	68	44	17	12	19	18	26	25	51	41	35	17	22	9	21	9
Tumor surface																
Smooth	187	53	37	11	60	18	90	21	110	45	72	21	44	11	45	13
Bloody	23	24	7	7	10	14	17	20	31	28	16	11	8	4	10	4
Palpable mass																
Present	11	13	5	6	4	7	14	13	15	15	3	5	6	2	3	4
Absent	199	64	39	12	66	25	93	28	126	58	85	27	46	13	52	13

BC, Breast Cancer; PND, Pathological Nipple Discharge; BCYH, Beijing Chao-Yang Hospital; HDH, Beijing Haidian District Maternal and Child Health Care Hospital; DLH, the Second Hospital of Dalian Medical University; HRH, Beijing Huairou Hospital; PGH, Beijing Pinggu Hosptial; YQH, Beijing Yanqing District Maternal and Child Health Care Hospital.

In the training set, bloody PND accounted for 35.1% (27/77) in the BC patient group versus 13.3% (28/210) in control patients (p<0.001). Ages were 46 (23-85) and 43 (19-80) years in the BC and control groups, respectively. Compared with non-BC patients, PND colors in BC patients were: colorless (n=2 vs. n=51), yellow (n = 48 vs. n=131), brown (n = 17 vs. n=18), and red (n = 10 vs. n=10) (p<0.001). There were 16.9% BC patients with palpable masses, whereas 5.2% of patients with palpable masses were pathologically proven as non-BC (p=0.002) (**Table 1**). In addition, irregular lesions that were visible by ductoscopy accounted for 57.1% (44/77) in the BC patient group versus 32.4% (68/210) in control patients (p<0.001). The detailed baseline characteristics in other test datasets and a study flowchart are shown in **Table 1** and **Figure 2**, respectively.

### Diagnostic performance of IDBCS

**Figure 3:**
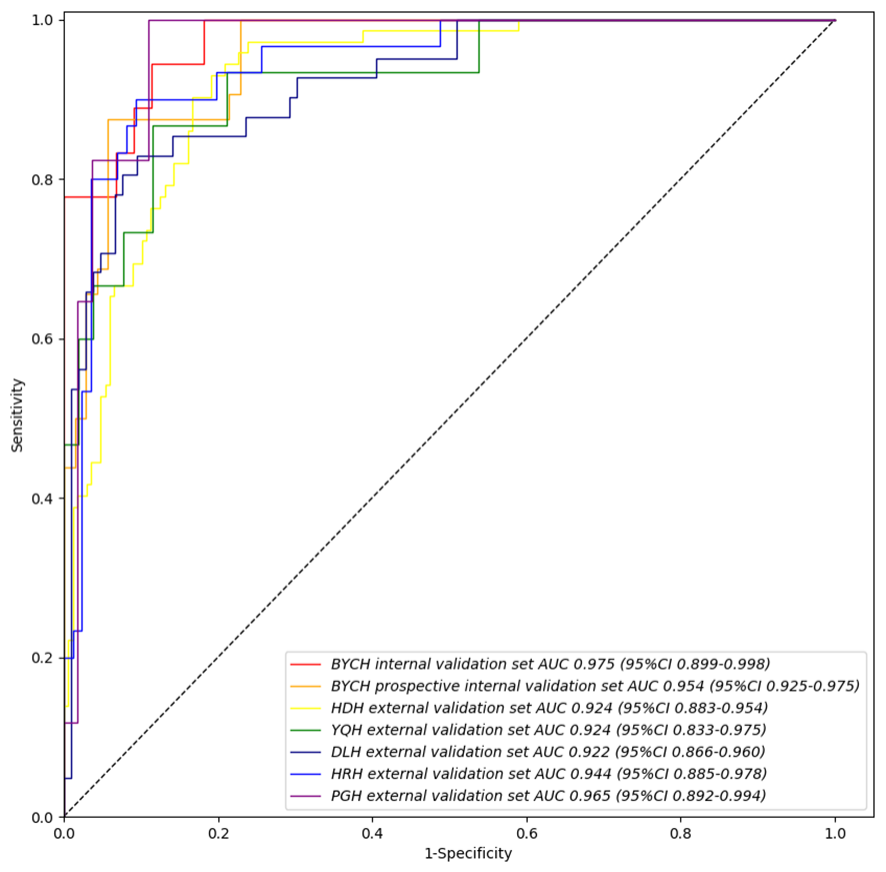
IDBCS’s performance on different validation datasets. The datasets contain BCYH internal validation set, BCYH prospective internal validation set, HDH external validation set, YQH external validation set, DLH external validation set, HRH external validation set, and PGH external validation set. The ROC curve and AUC were all calculated on the patient level, which has been clarified in equation (1).

In order to identify the most suitable base model for breast cancer diagnosis, the performances of Resnet, DenseNet, Inception and VGG were compared. Finally, we trained and assessed the performance of DenseNet as the best model in all seven validation sets (**Supplemental Figure 1**). We found that the Intelligent Ductoscopy for Breast Cancer Diagnostic System (IDBCS) had high performance in identifying BC patients. In internal and prospective BCYH validation datasets, diagnostic accuracies were 88.7% and 91.2%, respectively. In external validation datasets, accuracies were 84.2% for HDH, 86.6% for YQH, 87.8% for DLH, 89.7% for HRH, and 90.3% for PGH. The sensitivity and specificity of the novel IDBCS were >80% in the totality of validation datasets; its NPVs were higher than 90%, and PPVs were 66.7-76.7% (**Table 2**). IDBCS’ specificity and PPV were the lowest in DLH among all validation datasets. Elevated AUCs (0.922-0.965) suggested a great diagnostic performance for the IDBCS in the five validation datasets (**Figure 3**).

**Table 2:** Performance of IDBCS in different validation sets.

	BCYH validation		External validation set				
	Internal validation set	Prospective set	HDH	YQH	DLH	HRH	PGH
**AUC**	0.975 [0.899, 0.998]	0.954 [0.925, 0.975]	0.924 [0.883, 0.954]	0.924 [0.833, 0.975]	0.922 [0.866, 0.960]	0.944 [0.885, 0.978]	0.965 [0.892, 0.994]
**ACC (95%CI)**	88.71 [78.11, 95.34]	91.18 [83.91, 95.89]	84.17 [78.92, 88.55]	86.57 [76.03, 93.67]	87.76 [81.34, 92.58]	89.66 [82.63, 94.54]	90.28 [80.99, 96.00]
**SENS (95%CI)**	88.89 [65.29, 98.62]	84.38 [67.21, 94.72]	91.67 [82.74, 96.88]	80.00 [51.91, 95.67]	80.49 [65.13, 91.18]	86.67 [69.28, 96.24]	94.12 [71.31, 99.85]
**SPEC (95%CI)**	88.64 [75.44, 96.21]	94.29 [86.01, 98.42]	80.95 [74.19, 86.59]	88.46 [76.56, 96.65]	90.57 [83.33, 95.38]	90.70 [82.49, 95.90]	89.09 [77.75, 95.89]
**PPV (95%CI)**	76.19 [57.98, 88.13]	87.10 [72.04, 94.65]	67.35 [59.98, 73.95]	66.67 [47.48, 81.56]	76.74 [64.22, 85.85]	76.47 [62.34, 86.45]	72.73 [55.39, 85.14]
**NPV (95%CI)**	95.12 [84.02, 98.64]	92.96 [85.48, 96.73]	95.77 [91.30, 98.00]	93.88 [84.72, 97.70]	92.31 [96.53, 95.73]	95.12 [88.65, 97.99]	98.00 [87.95, 99.70]

95% confidence intervals are included in brackets. AUC, area under the receiver operating characteristic curve; ACC, accuracy; SENS, sensitivity; SPEC, specificity; PPV, positive predict value; NPV, negative predict value. BCYH, Beijing Chao-Yang Hospital; HDH, Beijing Haidian District Maternal and Child Health Care Hospital; DLH, the Second Hospital of Dalian Medical University; HRH, Beijing Huairou Hospital; PGH, Beijing Pinggu Hosptial; YQH, Beijing Yanqing District Maternal and Child Health Care Hospital.

Furthermore, this model was extended to be compatible with two groups of tasks for cancer subtype prediction. The numbers of lesions in the two categories were 170 (ductal carcinoma in situ, DCIS) and 40 (invasive breast carcinoma, IBC), respectively. The overall accuracies in differentiating the two groups ranged from 50.0% to 70.3% in all validation datasets (**Supplemental Figure 2**). The model performed well in distinguishing benign from malignant tumors, while showing lower potential in differentiating cancer subtypes (DCIS vs. IBC).

### Performance of IDBCS versus endoscopists

**Table 3** summarizes the results of the IDBCS and 9 endoscopists for differentiating between 511 (151 [29.5%] cancer and 360 [70.5%] control images) in the prospective validation dataset. IDBCS detected BC with an accuracy of 0.912 (95%CI 0.839-0.959). In comparison, expert endoscopists had markedly reduced accuracy (0·726, 95%CI 0.672-0.775; p<0.001), as well as competent endoscopists (0.699, 95%CI 0.645-0.750; p<0.001) and trainee endoscopists (0.702, 95%CI 0.648-0.753; p<0.001). Moreover, there was no statistical difference in specificities. However, sensitivities, PPVs and NPVs for all three categories of endoscopists were significantly lower than those of IDBCS (p<0.001).

**Table 3:** Performance of IDBCS versus human endoscopists in identifying breast cancer in a randomly selected subset of patients (n=102) from the prospective validation group.

	ACC (%)	SENS (%)	SPEC (%)	PPV (%)	NPV (%)
**IDBCS**	91.18 [83.91, 95.89]	84.38 [67.21, 94.72]	94.29 [86.01, 98.42]	87.10 [72.04, 94.65]	92.96 [85.48, 96.73]
**Expert endoscopist**	72.55 [67.18, 77.47]	25.00 [16.72, 34.88]	94.29 [90.23, 97.01]	66.67 [51.09, 79.29]	73.33 [70.92, 75.62]
**Competent endoscopist**	69.94 [64.46, 75.02]	18.75 [11.51, 28.01]	93.33 [89.07, 96.31]	56.25 [40.03, 71.23]	71.53 [69.40, 73.58]
**Trainee endoscopist**	70.26 [64.80, 75.33]	14.58 [8.21, 23.26]	95.71 [92.02, 98.02]	60.87 [41.10, 77.62]	71.03 [69.19, 72.79]

95% confidence intervals are included in brackets.

ACC, accuracy; SENS, sensitivity; SPEC, specificity; PPV, positive predict value; NPV, negative predict value.

Among endoscopists, expert endoscopists had significantly higher sensitivity than the competent (0.250, 95%CI 0.167-0.349 vs. 0.188, 95%CI 0.115-0.280; p<0.001) and trainee (0.146, 95%CI 0.082-0.233; p<0.001) endoscopist groups. Other diagnostic indicators such as accuracy, specificity, PPV and NPV were similar among these endoscopist groups. Here we also attached our model and the data of previous articles for comparison in **Supplemental Table 1**.

### Visual explanation of the decision made by the IDBCS

**Figure 4:**
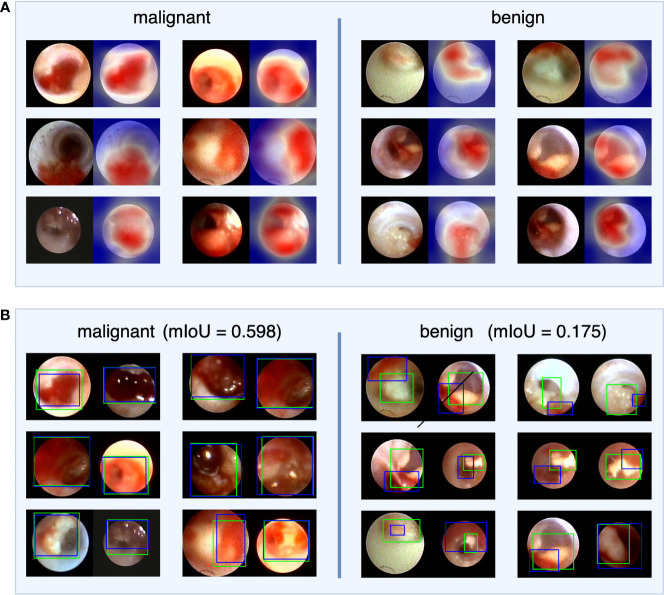
The visual explanation of IDBCS. The part **(A)** of this figure shows the decision-supported regions. For each image pair, the left image is the original ductoscopy image and the right image is the heatmap. In the heatmap, the red regions are the important regions that support the diagnosing result of IDBCS. In part **(B)** of this figure, the blue rectangle represents the annotated hemorrhage area and the green rectangle represents the decision-supported area. As we can see in this figure, the decision-supported area and the hemorrhage area has a higher mIoU in malignant tumors. This phenomenon means that the hemorrhage is an important reason for malignant tumor diagnosing.

To investigate IDBCS interpretability, we used the Score-CAM (23) algorithm to identify important regions on a single tumor image that supports the algorithm’s decision. The heatmap in **Figure 4** highlights the important regions in red and the less important ones in blue. In the heatmaps, the important regions in malignant tumors were often accompanied by hemorrhage. Therefore, we designed a study to further investigate the correlation between hemorrhage and IDBCS interpretability. According to the study results, the average intersection over union (IoU) value between the areas of malignant tumors and those of hemorrhage was 0.598, which was higher than the average IoU (0.175, p<0.001) in benign intraductal lesions.

### Diagnosing system based on IDBCS

The developed IDBCS with DenseNet was able to analyze and process as many as 31 images per second (32ms per image) on average for real-time ductoscopy diagnosis using Nvidia Tesla T4 GPU. We also test the diagnosing speed of IDBCS under the other backbones: VGG (250 images per second), ResNet (70 images per second), and Inception (43 images per second). Our IDBCS can meet real-time requirements under all the above four backbones. Although the diagnosing speeds under VGG, ResNet and Inception are faster, IDBCS with DenseNet can achieve higher diagnostic accuracy. In addition, we apply the IDBCS algorithm to the current ductoscopy system. Specifically, the ductoscopy images were transmitted to our computing server and then the diagnosing result given by IDBCS will be shown on the monitor in real-time. The demo video in our supplementary file shows an example of IDBCS real-time diagnosing.

## Discussion

Here, a deep learning model was utilized for constructing an artificial intelligence-based BC diagnostic system, termed IDBCS, which was trained and validated with 11305 endoscopy images acquired in 1072 individuals in 6 hospitals with diverse experiences and amounts of pathological nipple discharge cases. The IDBCS had high accuracy, sensitivity, and specificity for BC detection in retrospective and prospective observational settings. This study first performed artificial intelligence-guided breast cancer detection according to PND endoscopic images. We demonstrated that the IDBCS was superior to endoscopists in differentiating malignancy from benignity for intraductal tumors. Additionally, an evidence-based visual explanation derived from the IDBCS was provided, which may be used routinely in the clinic.

PND is one of the three major symptoms of breast disease. Malignancy rates between 1% and 23% have been reported in PND cases (24). To characterize PND, mammography and ultrasonography are frequently employed, but the results are often negative (25). Moreover, breast MRI does not add much (26). Furthermore, galactography and cytological analysis of nipple discharge have low sensitivities, and generally do not identify the associated pathology (27). Currently, ductoscopy that visualizes intraductal lesions as a minimally invasive procedure is the most commonly utilized imaging modality for evaluating PND. However, the learning curve of ductoscopy is long, and its routine use in PND cases is limited in clinic. A recent meta-analysis (28) showed pooled sensitivity and specificity for ductoscopy of 50% (36-64%) and 83% (81-86%), respectively, which is similar to the detection ability of endoscopists in this study. Therefore, novel tools for helping endoscopists differentiate benign tumors from cancerous ones on ductoscopy images are urgently needed.

DL methods have been predominantly utilized for image processing. An advantage of DL models is the possibility of immediate and consistent data reporting, thus reducing the workload, as well as inconsistencies, and misdiagnoses. Additionally, it overcomes the inherent limitations of doctors, including perceptual bias and visual fatigue (29). Besides, the DL model-associated visual display further provides evidence-based classification to help endoscopists interpret the images. Recently, DL has been broadly employed in endoscopy. Previously published reports have developed DL models based on gastroscopy and colonoscopy images for identifying gastrointestinal tumors (10–12). Li Caofeng et al. developed an endoscopic image-based nasopharyngeal cancer detection model to diagnose nasopharyngeal cancer (30). In this study, the IDBCS was designed for visual diagnosis of breast cancer including the largest amount of ductoscopy images from routine white-light imaging methods.

The IDBCS could decrease the reliance upon the endoscopists’ expertise for visual breast cancer diagnosis and increase diagnostic consistency. A strength of this work is that it included diverse noncancerous pathologies (i.e., intraductal papilloma, inflammation, and ductal hyperplasia) in the control group and utilized pathological assessment as the gold standard. Consequently, IDBCS could learn noncancerous properties that usually complicate breast cancer diagnosis, likely enhancing model performance and avoiding verification bias. The IDBCS underwent training and fine-tuning with multiple images acquired in individuals diagnosed at our center over 3 years, with encouraging performance in a short learning time, indicating IDBCS ‘learns’ efficiently and has high productivity. The overall accuracy of the novel IDBCS system was 91.2%, which surpasses the value reported for endoscopists’ diagnosis (31, 32). At our center, the IDBCS system also had markedly elevated accuracy and specificity compared with endoscopists at various levels of expertise. The above findings suggest the IDBCS system has great potential for improving the diagnosis of breast cancer with PND.

Retrospective trials have assessed the association between nipple discharge and BC. Ye Han (33) reported the endoscopic characteristics of bloody discharge, morphology and a wide tumor base independently predict breast cancer with PND. A meta-analysis (34) showed that bloody nipple discharge is a predictive factor of BC risk among diverse discharge colors. However, few reports focused on the interpretability between bloody nipple discharge and BC risk. In the current study, the important regions of malignant tumors were often accompanied by hemorrhage based on the Score-CAM algorithm. To our knowledge, marked redness could be associated with dilated tumor vessels, and the abrupt rupture and necrosis of the local tissue are associated with tumor-induced fibrosis. Currently, IDBCS is included in the routine endoscopic workflow with real-time evaluation at BCYH, providing free-access to deep learning-aided breast cancer screening and diagnosis.

There were several limitations in this study. Firstly, the number of enrolled participants was not large. To overcome this limitation of small sample size, extensive data augmentation was carried out in model training. In addition, the proposed model can only differentiate malignant tumors from benign ones. Although two BC types were included, i.e., ductal carcinoma *in situ* and invasive BC, the overall accuracy of the model in differentiating them was poor due to their small sample sizes. Future investigation will expand the new model to further determine whether the malignant lesions detected are DCIS or IBC. Furthermore, the training and test datasets mostly included northern Chinese cases, and IDBCS’ performance in other ethnicities is unknown. Finally, other clinical data, e.g., age, PND characteristics and tumor morphology were not considered. Therefore, a multi-source imaging diagnosis model should be established for clinical application in breast cancer detection based on deep learning.

Overall, an efficient real-time AI system using ductoscopy images was developed for breast cancer detection in the real world. The IDBCS system had excellent performance in BC detection in independent validation datasets. However, since this study was limited by a poor diagnostic value for different BC subtypes, multicenter prospective validation with larger datasets is warranted for high-level evidence in breast cancer subtype analysis.

## Data availability statement

The raw data supporting the conclusions of this article will be made available by the authors, without undue reservation.

## Ethics statement

Written informed consent was obtained from the individual(s) for the publication of any potentially identifiable images or data included in this article.

## Author contributions

FX, CZhu, JLiu, and HJ contributed to the study concept and design. ZW, LZ, HG, ZM, YGa, YGu, and XL contributed to acquisition of data. YZL, ML, GS, HL, YSL, CZha, JC, JLi, JLiu, and HJ contributed to analysis and interpretation of data. All authors contributed to writing, reviewing, and approval of the final version of this work.

## Supplementary material

Supplementary Video 1 Instruction of IDBCS real-time diagnostic video. This video shows the real-time diagnostic process of IDBCS. The diagnostic results are marked in the upper left corner of the video, with 0 for benign tumor and 1 for malignant tumor. In this video, all tumors appeared are benign. They appeared at seconds 11, 18, 21, 24, 54, and 84 of the video, and were all successfully diagnosed by the algorithm.
