# Supplementary material for: Deep learning for real-time detection of breast cancer presenting pathological nipple discharge by ductoscopy
Source: Front Oncol. 2023 Mar 22;13:1103145. doi: 10.3389/fonc.2023.1103145 (PMC10073663; doi:10.3389/fonc.2023.1103145)
Supplement: Supplementary file 2 [file DataSheet_1.docx]

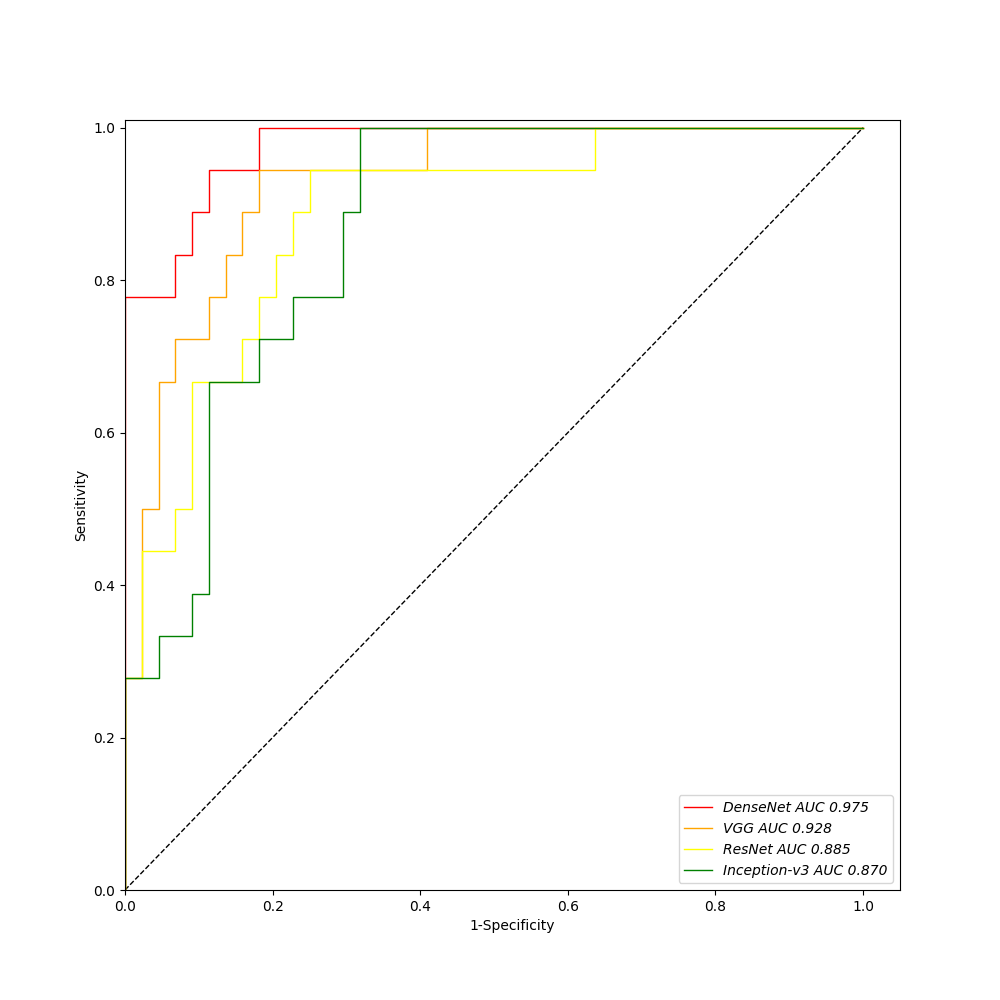


**Supplemental figure 1.** The figure shows different models’ performance on the BCYH internal validation set. The models contain DenseNet, VGG, ResNet, and Inception-v3. The ROC curve and AUC were all calculated on the patient level, which means every patient was seen as a single sample.


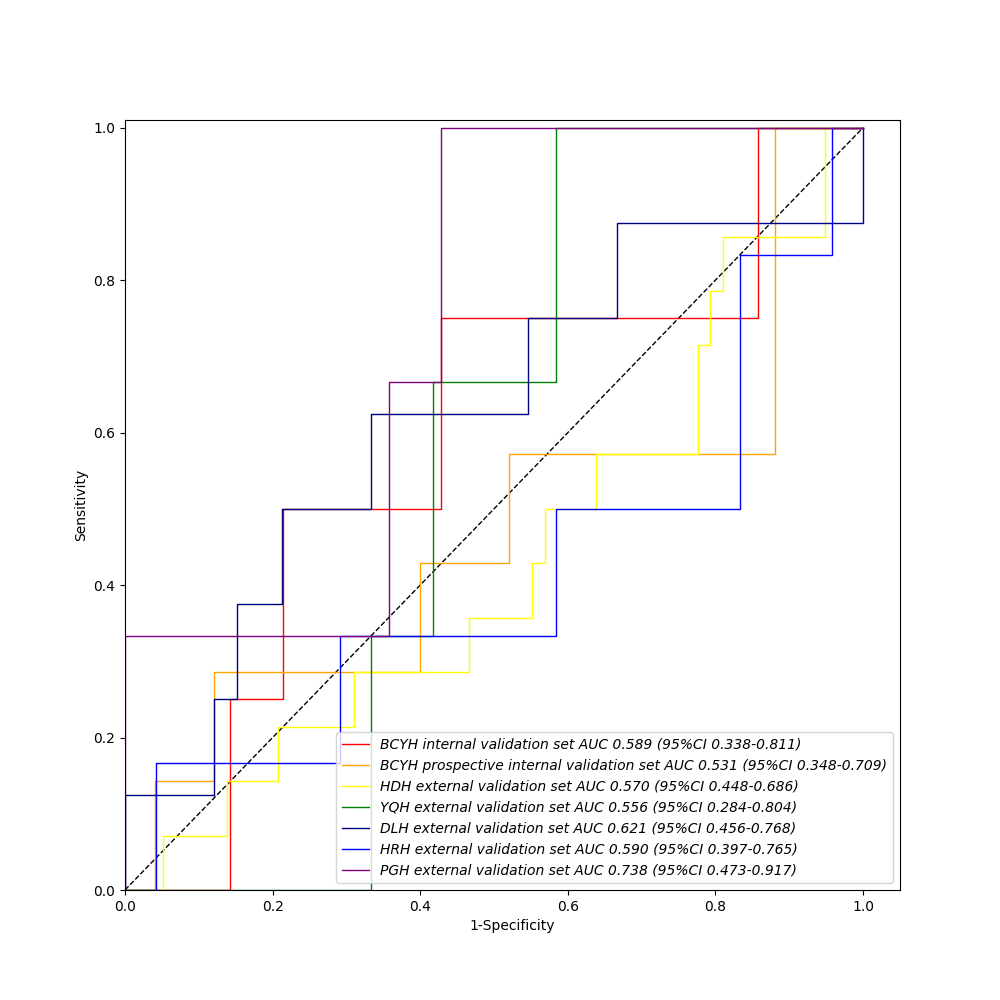


**Supplemental figure 2.** The figure shows IDBCS’s performance in the classification of carcinoma in situ and invasive carcinoma. The ROC curve and AUC were all calculated on the patient level, which means every patient was seen as a single sample.

Table 1. Performance of IDBCS versus the data of previous articles in diagnosing breast cancer by using ductoscopy.

|  | **Accuracy** | **Sensitivity** | **Specificity** | **PPV** | **NPV** |
| --- | --- | --- | --- | --- | --- |
| **IDBCS model** | 0.91[0.84,0.96] | 0.84[0.67,0.95] | 0.94[0.86,0.98] | 0.87[0.72,0.95] | 0.93[0.85,0.97] |
| **Yilmaz et al** | 0.82[0.60,0.95] | 0.94[0.71,1.00] | 0.40[0.05,0.85] | 0.84[0.60,0.97] | 0.67[0.09,0.99] |
| **Simpson et al** | 0.79[0.64,0.91] | 0.67[0.09,0.99] | 0.81[0.64,0.92] | 0.22[0.03,0.60] | 0.97[0.83,1.00] |
| **Denewer et al** | 0.75[0.62,0.86] | 0.18[0.02,0.52] | 0.90[0.77,0.97] | 0.33[0.04,0.78] | 0.81[0.67,0.91] |
| **Cyr AE et al** | 0.71[0.62,0.79] | 0.67[0.30,0.93] | 0.71[0.61,0.79] | 0.16[0.06,0.31] | 0.96[0.90.0.99] |
| **Moncrief RM et al** | 0.83[0.71,0.92] | 0.14[0.00,0.58] | 0.92[0.81,0.98] | 0.20[0.01,0.72] | 0.89[0.77,0.96] |

1.Yilmaz R, Bender O, Yabul FC, et al. Diagnosis of Nipple Discharge: Value of Magnetic Resonance Imaging and Ultrasonography in Comparison with Ductoscopy. Balkan Med J. 2017;34(2):119-26.

2.Denewer A, El-Etribi K, Nada N, et al. The role and limitations of mammary ductoscope in management of pathologic nipple discharge. Breast J. 2008;14(5):442-9.

3.Moncrief RM, Nayar R, Diaz LK, et al. A comparison of ductoscopy-guided and conventional surgical excision in women with spontaneous nipple discharge. Ann Surg. 2005;241(4):575-81.

4.Simpson JS, Connolly EM, Leong WL, et al. Mammary ductoscopy in the evaluation and treatment of pathologic nipple discharge: a Canadian experience. Can J surgery Journal Can Chir. 2009;52(6): E245-8.

5.Cyr AE, Margenthaler JA, Conway J, et al. Correlation of ductal lavage cytology with ductoscopy-directed duct excision histology in women at high risk for developing breast cancer: a prospective, single-institution trial. Ann Surg Oncol. 2011;18(11):3192-7.
